# Knockdown of ELMO3 Suppresses Growth, Invasion and Metastasis of Colorectal Cancer

**DOI:** 10.3390/ijms17122119

**Published:** 2016-12-16

**Authors:** Hui-Yun Peng, Qiong-Fang Yu, Wei Shen, Cheng-Ming Guo, Zhen Li, Xiao-Yan Zhou, Nan-Jin Zhou, Wei-Ping Min, Dian Gao

**Affiliations:** 1 Department of Pathogen Biology and Immunology, Medical College of Nanchang University, Nanchang 330006, China; huiyun.peng@foxmail.com (H.-Y.P.); lizhen8139091@163.com (Z.L.); weiping.min@gmail.com (W.-P.M.); 2 Department of Gastroenterology and Hepatology, Second Affiliated Hospital of Nanchang University, Nanchang 330006, China; qiongfangyu@yeah.net (Q.-F.Y.); chengming.guo@foxmail.com (C.-M.G.); 3 Department of Gastrointestinal Surgery, Second Affiliated Hospital of Nanchang University, Nanchang 330006, China; shenweimd@hotmail.com; 4 Department of Pathophysiology, Medical College of Nanchang University, Nanchang 330006, China; zhouxiaoyan@ncu.edu.cn; 5 Institute of Molecular Medicine, Jiangxi Academy of Medical Sciences, Nanchang 330006, China; jxznj@163.com

**Keywords:** engulfment and cell mobility, ELMO, proliferation, invasion, metastasis, colorectal cancer

## Abstract

The engulfment and cell motility (ELMOs) family of proteins plays a crucial role in tumor cell migration and invasion. However, the function of ELMO3 is poorly defined. To elucidate its role in the development and progression of colorectal cancer (CRC), we examined the expression of ELMO3 in 45 cases of paired CRC tumor tissues and adjacent normal tissues. Furthermore, we assessed the effect of the knockdown of ELMO3 on cell proliferation, cell cycle, migration, invasion and F-actin polymerization in HCT116 cells. The result shows that the expression of ELMO3 in CRC tissues was significantly increased in comparison to the adjacent normal colorectal tissues. Moreover, this overexpression was associated with tumor size (*p* = 0.007), tumor differentiation (*p* = 0.001), depth of invasion (*p* = 0.009), lymph node metastasis (*p* = 0.003), distant metastasis (*p* = 0.013) and tumor, node, metastasis (TNM)-based classification (*p* = 0.000). In in vitro experiments, the silencing of ELMO3 inhibited cell proliferation, invasion, metastasis, and F-actin polymerization, and induced Gap 1 (G1) phase cell cycle arrest. Our study demonstrates that ELMO3 is involved in the processes of growth, invasion and metastasis of CRC, and could be used a potential molecular diagnostic tool or therapy target of CRC.

## 1. Introduction

Colorectal cancer (CRC) is one of the most common human malignancies and remains the third leading cause of malignancy-related mortality in Western countries [1,2,3]. With the rapid growth of the world economy, the incidence of CRC in some developing countries has risen sharply over the past decades [4,5]. Approximately 40%–50% of all CRC patients develops metastatic disease after resection of the primary CRC [6]. Tumor invasion and metastasis weaken the effect of treatment and are a major reason in the death of CRC patients.

Chemotactic movement is a specific form of cell migration [7,8]. It consists of three basic cell processes: chemoattractant gradient sensing, actin-dependent cell polarization, and actin-mediated cell motility [9,10]. The ELMOs family is a highly evolutionarily conserved protein family that plays a crucial role in cytoskeleton rearrangements during phagocytosis, cellular migration and chemotaxis [11]. These proteins were originally identified as orthologs of *Caenorhabditis elegans* CED-12 [11,12] and Dictyostelium discoideum ELMOA-F [13]. In humans, this family consists three members: ELMO1, ELMO2 and ELMO3. The main protein structure of ELMOs is highly conserved and includes the ras GTPase-binding domain (RBD), ELMO inhibitory domain (EID), ELMO domain, PH domain, ELMO autoregulatory domain (EAD) and the extreme C-terminal proline-rich motifs [14,15]. Previous reports have revealed that ELMO1 and -2 are aberrantly expressed in a variety of human malignant tumors including primary gliomas [16,17], breast cancer [18], liver cancer [19], ovarian cancer [20,21], esophageal cancer [22] and rhabdomyosarcoma [23], and are closely related to the development, invasion and metastasis of tumors and play key roles in related signal regulating processes [16,20,24,25,26,27,28,29]. The ELMO1 and Dock proteins form a stable complex, which can be regulated by CXCR4 and directly interacts with the G protein α or β subunit to regulate Rac1 activation in response to chemoattractant G protein-coupled receptor activation. The activation of Rac promotes the growth of the dendritic actin-cytoskeleton, and controls cell migration during multiple processes, such as the engulfment of apoptotic cells, phagocytosis, tumor invasion, myoblast fusion and angiogenesis [18,25,26]. The ELMO2/Dock complex contributes to both integrin- and cadherin-based adhesions, which are involved in the transition of cells from migration to strong cell–cell adhesion [30].

However, the role and function of ELMO3 are rarely characterized due to its low or absent expression in most cancer tissues or cell lines [31]. The expression of ELMO3 has only been reported in lung cancer with distant metastasis [32,33,34,35] and colon cancer cell lines [36]. In lung cancer, the overexpression of ELMO3 coincides with the hypomethylation of its promoter CpG island. In CRC cells, the ELMO3 promoter can be activated by CDX2 and SP1. Generally, the function of ELMO3 has not been directly elucidated.

In this study, we hypothesized that ELMO3 could play a key role in the cell growth, invasion and metastasis of colorectal cancer. Therefore, we analyzed the relationship between the aberrant expression of ELMO3 in CRC tissues and clinicopathological characteristics. Furthermore, we investigated the function of ELMO3 in the proliferation, cell cycle regulation, invasion and metastasis of CRC HCT116 cells. The results reveal that the overexpression of ELMO3 in CRC tissues is associated with the formation of metastases. Moreover, silencing ELMO3 significantly downregulated the proliferation, invasion and metastasis and inhibited the F-actin polymerization of HCT116 cells.

## 2. Results

### 2.1. Expression of ELMO3 Was Aberrant in Human Colorectal Cancer (CRC) Tissues

To clarify the relationship between the expression of ELMO3 in CRC tissue and clinicopathological characteristics, we examined the mRNA and protein levels of ELMO3 in paired CRC tumor tissues and adjacent normal tissues.

**Figure 1:**
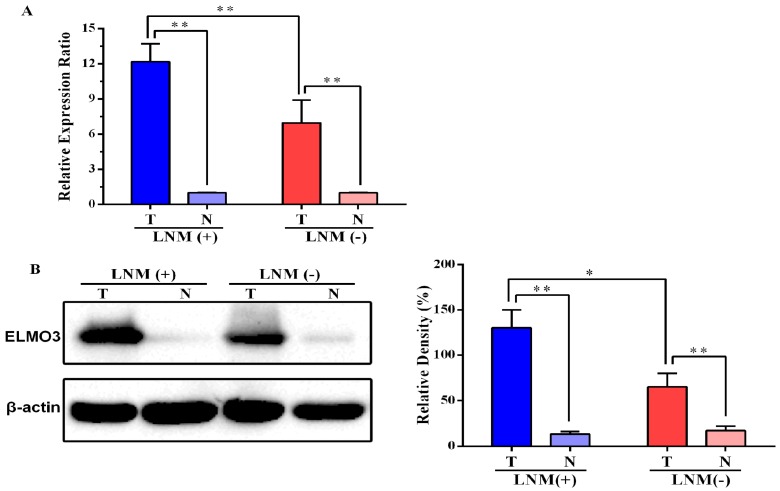
The mRNA and protein levels of ELMO3 in primary colorectal cancer (CRC) tissues and adjacent normal tissues: (**A**) Real-time quantitative PCR was used to detect the ELMO3 mRNA expression level in primary tumors (T) and matched adjacent normal tissues (N) in CRC patients. LNM (+): patients with lymph node metastasis; LNM (−): patients without lymph node metastasis. β-actin was used as a reference gene to normalize ELMO3 expression. The 2^–ΔΔ*C*t^ method was used to calculate the relative expression ratio. Error bars indicate standard deviation (SD) and (**B**) Western blot analysis was used to determined protein level of ELMO3. The statistical analysis was performed by Analysis of variance (ANOVA). * *p* < 0.05, ** *p* < 0.01.

The result of real-time quantitative PCR (qPCR) analysis reveals that the mRNA level of ELMO3 in CRC tissues was significantly higher than that in adjacent normal tissues (*p* = 0.000, Figure 1A). Moreover, for those CRC patients with lymph node metastasis, the mRNA level of ELMO3 in CRC tissues was markedly higher than that in CRC tissues from patients without lymph node metastasis (*p* = 0.000).

The result of the Western blot analysis was similar to that of the qPCR analysis. CRC tumor tissues expressed markedly higher level of ELMO3 protein than paired adjacent normal tissues (*p* = 0.000, Figure 1B). Furthermore, the protein level of ELMO3 in patients with lymph node metastasis was also significantly higher than that in patients without lymph node metastasis (*p* = 0.011).

### 2.2. The Correlation between ELMO3 Expression and Clinicopathological Characteristics

**Figure 2:**
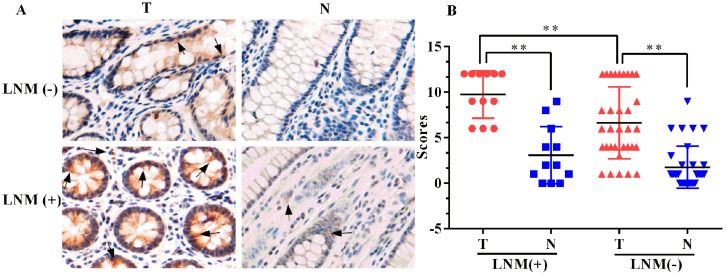
Immunolocalization of ELMO3 in primary CRC tumor tissues (T) and adjacent normal tissues (N): (**A**) The examination was carried out using immunohistochemical staining. LNM (+): patients with lymph node metastasis; LNM (−): patients without lymph node metastasis. Arrows indicate the positive staining. The representative photographs are shown at 200× magnification and (**B**) The statistical analysis was performed using an ANOVA. ** *p* < 0.01.

The expression of ELMO3 in CRC tissues was also detected by immunohistochemistry (IHC) analysis. The immunoreactivity of the ELMO3 protein was mainly observed in the cell cytoplasm and cell membrane. Stronger staining of ELMO3 was detected in cancer cells with lymph node metastasis than in cancer cells without lymph node metastasis (*p* = 0.003, Figure 2). As shown in Table 1, the positive expression rate of the ELMO3 protein in the tumor tissues (88.9%) was higher than that in the adjacent normal tissues (37.78%) (*p* = 0.000).

The correlations between the expression of ELMO3 and the clinicopathological parameters of CRC were further analyzed. As indicated in Table 2, positive immunostaining of ELMO3 was significantly correlated with tumor size (*p* = 0.007), pathologic differentiation degree (*p* = 0.001), depth of invasion (*p* = 0.009), lymph node metastasis (*p* = 0.003), distant metastasis (*p* = 0.013) and TNM stage (*p* = 0.000). However, no significant correlation was found between ELMO3 expression and gender (*p* = 0.883) or age (*p* = 0.094).

### 2.3. Knockdown of ELMO3 Inhibits Cell Proliferation of HCT116 Cells

**Figure 3:**
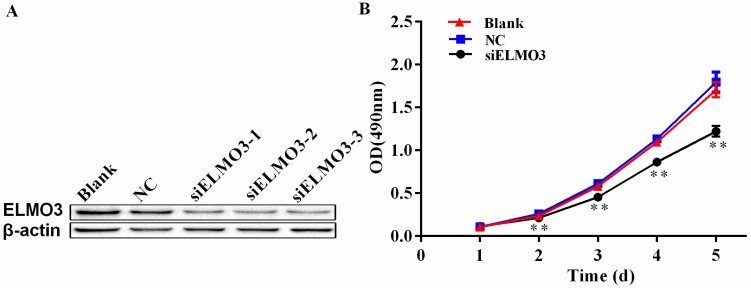
Knockdown of ELMO3 suppressed the proliferation of HCT116 cells. (**A**) The inhibition efficiency of the candidate ELMO3 siRNAs was determined by Western blot analysis. β-actin was used as a loading control. The result shows that siELMO3-3 had the highest silencing efficiency; (**B**) An MTS assay determined the effect of silencing ELMO3 on the proliferation capacities of HCT116 cells. NC represents negative control group. Values represent the mean ± SD of the absorbance at various time points (*n* = 6, analysis of variance of factorial design). ** *p* < 0.01.

The aberrant expression of ELMO3 in CRC indicates that ELMO3 possibly plays a critical role in the development and progression of CRC. Thus, we explored the function of ELMO3 in cell proliferation. The expression of ELMO3 was first examined in six human CRC cell lines, including HCT116, LoVo, COLO205, HT29, SW480 and SW620 by RT-PCR analysis and Western blot analysis (Figure S1). The results indicate that ELMO3 was expressed in all six of the cell lines. A relatively high level of ELMO3 protein expression was observed in the aggressive or poorly differentiated HCT116 and LoVo cell lines. HCT116 cells were used for the subsequent experiments. The efficiency of knockdown ELMO3 was detected by Western blot analysis. From this analysis, a ELMO3-specific siRNA (siELMO3) with the target sequence 5’-GCGGAACGUGGUGAAGAUUTT-3’ (sense) and 5’-AAUCUUCACCACGUUCCGCTT-3’ (antisense) was found to have the highest knockdown effect (81.5%) and used in the following experiments (Figure 3A).

The effect of ELMO3 silencing on HCT116 cell growth was assessed by 3-(4,5-dimethylthiazol-2-yl)-5-(3-carboxymethoxyphenyl)-2-(4-sulfophenyl)-2*H*-tetrazolium (MTS) assay. HCT116 cells were either transfected with siELMO3 or a negative control siRNA as NC, or left with untransfected as a blank control, and the cells were then incubated for 1–5 days. As shown in Figure 3B, the proliferation of the siELMO3-transfected cells was significantly inhibited when compared with that of the blank and negative control groups after two days of treatment. In the following three days, the difference increased.

### 2.4. Knockdown of ELMO3 Induces G1 Cell Cycle Arrest

**Figure 4:**
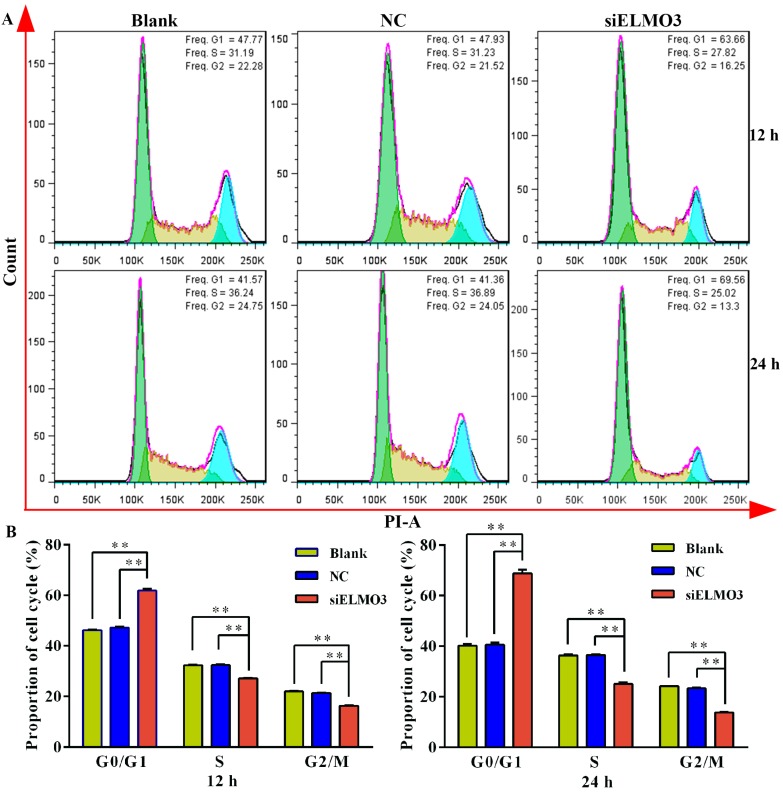
Knockdown of ELMO3 arrested the cell cycle of HCT116 cells at G1 phage. (**A**) The cell cycle distribution of HCT116 cells was detected by flow cytometry and (**B**) Statistical analysis was performed by ANOVA. ** *p* < 0.01.

To investigate whether silencing ELMO3 could induce cell cycle perturbation in HCT116 cells, the cell cycle was analyzed by flow cytometry using propidium iodide (PI) DNA staining. The results reveal that after 12 h of siELMO3 treatment, the fraction of cells in the G1 phase was significantly increased (63.66% compared to 47.93% in NC cells) (Figure 4), whereas the proportion of cells in the S phase (27.82% compared to 31.23% in NC cells) and in the G2/M phase (16.25% compared to 21.52% in NC cells) declined. By 24 h, a concomitant increase in the fraction of cells in the G1 phase and a lower percentage of cells in the S and G2/M phases were detected. These results indicate that siELMO3 treatment significantly induced G1 cell cycle arrest.

### 2.5. Knockdown of ELMO3 Supresses Cell Invasion and Metastasis

**Figure 5:**
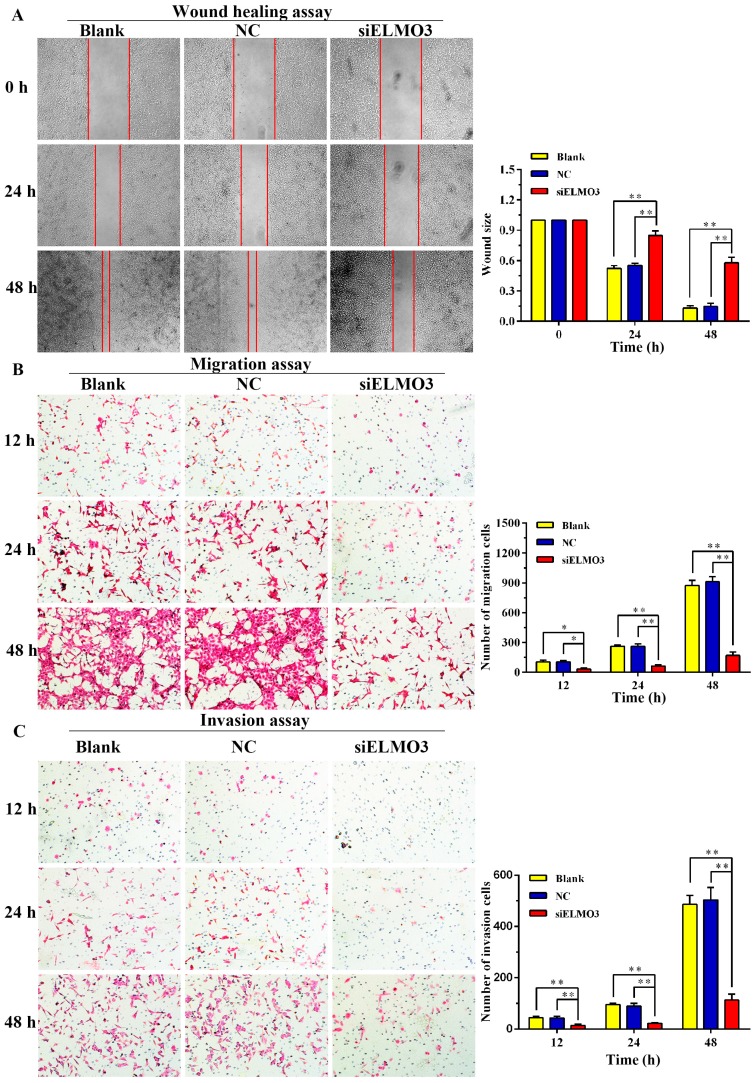
Knockdown of ELMO3 inhibited the migration and invasion of HCT116 cells. (**A**) The wound healing assay was carried out to examine the cell motility rates; (**B**) The Transwell assay was performed to assess cell migration potential; and (**C**) The matrigel Transwell assay was performed to assess cell invasion potential. The representative photographs are shown at 40× magnification. The statistical analysis was performed using ANOVA. * *p* < 0.05, ** *p* < 0.01.

Cell migration and invasion are important processes of tumor development and metastasis. Thus, we assessed the effect of ELMO3 on migration and invasion in HCT116 cells using both a wound healing assay and a transwell assay. As shown in Figure 5A, the silencing of ELMO3 suppressed the wound healing capacity at 24 h, which became evident by 48 h when compared with blank and NC groups. In addition, the transwell assay also reveals a similar result. Cell migration was significantly inhibited in the ELMO3 knockdown group. Compared with the blank and NC groups, a fewer number of siELMO3 cells were found on the filter surface (Figure 5B).

A Transwell assay was further employed to examine the effect of silencing ELMO3 on the invasion ability of HCT116 cells. As shown in Figure 5C, the invasion number of siELMO3 group was remarkably decreased compared with the blank and NC groups following treatment for 12, 24 and 48 h (*p* = 0.002, *p* = 0.000 and *p* = 0.000, respectively), which suggests that the downregulation of ELMO3 suppressed the mobility and migration of HCT116 cells.

### 2.6. Knockdown ELMO3 Supresses the F-Actin Polymerization of HCT116 Cells

**Figure 6:**
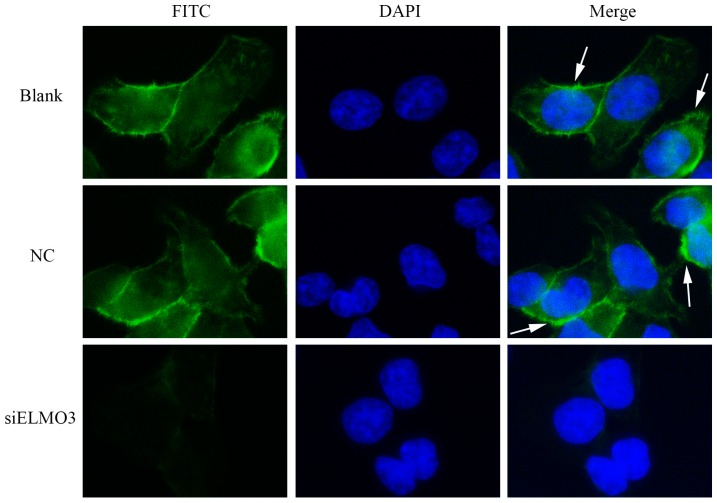
Knockdown of ELMO3 inhibited the F-actin polymerization of HCT116 cells. The cytoskeleton and nuclei were stained with FITC-phalloidin and DAPI. The fluorescent signals of F-actin, labeled **white** arrows, were detected on the plasma membrane in the NC and Control groups. The representative photographs are shown at 400× magnification.

Actin microfilaments (F-actin) are crucial components of the cytoskeleton, which is responsible for the shape and movement of eukaryotic cell. During the movement of cells, the polymerization of actin monomers into polarized F-actin plays a pivotal role [37]. To observe the effect of silencing ELMO3 on the organization of the cytoskeleton, HCT116 cells were stained with fluorescein isothiocyanate-phalloidin (FITC-phalloidin). Then, the cells were counterstained with 4’,6-diamidino-2-phenylindole (DAPI) to visualize cell nuclei. The cells in the blank and NC groups possessed an abundant content of actin with regular structure (Figure 6). In contrast, the actin assembly in the siELMO3 group was altered dramatically. The cells had fewer filaments. However, the nuclear morphology did not reveal any obvious change when compared with that in the blank and NC groups. This result indicates that the knockdown of ELMO3 inhibited the actin polymerization of HCT116 cells, which causes a delay in migration.

## 3. Discussion

The ELMO protein family members play important roles in regulating actin-mediated processes such as chemotaxis and phagocytosis [13]. However, the role and regulating mechanism of EMLO3 remains unclear compared with those of ELMO1 and ELMO2, which present unique and overlapping functions in different types of cancer [16,17,18,19,20,21,22,23]. In our current study, we observed high mRNA and protein levels of ELMO3 in CRC tissues and cell lines, which differs from that in many other cancers, including breast cancer, kidney cancer, hepatic cancer, etc. This finding indicates that ELMO3 possibly plays a crucial and distinct role in the development and progression of CRC. Furthermore, ELMO3 was expressed more highly in CRC tumor tissues from patients with lymphatic node metastasis than that in CRC tumor tissues from patients without lymphatic node metastasis, which shows that ELMO3 is possibly involved in colorectal cancer metastasis. More evidence was acquired by IHC analysis, which revealed that the expression of ELMO3 was positively correlated with the pathologic differentiation degree, depth of invasion, lymph node metastasis, distant metastasis and TNM stage. The findings are consistent with a previous study of lung cancer [32], where the expression of ELMO3 was significantly upregulated in the tissues and serum from non-small cell lung cancer (NSCLC) patients, significantly associated with many clinicopathological characteristics and can be used as a potential diagnostic and prognostic marker. It further indicates that ELMO3 may serve an important function in the development and progression of CRC.

Silencing ELMO3 markedly suppressed the proliferation of HCT116 cells, which shows that ELMO3 plays an important role in cell growth. In breast cancer, a similar study also revealed that the knockdown of ELMO2 diminishes breast cancer cell proliferation, which is regulated by the receptor tyrosine kinase Axl through phosphorylating a conserved carboxyl-terminal tyrosine residue of ELMO2 [38]. However, because no ELMO3 expression was detected in breast cancer, it is difficult to speculate whether ELMO3 acts through a similar mechanism in regulating colorectal cell growth. Moreover, a previous study showed that the regulation of cell proliferation often occurs during the G1 phase of cell division cycle [39]. Our result of flow cytometric analysis shows that the knockdown of ELMO3 induced cell cycle arrest at G1 phase, which indicated that the inhibition of cell proliferation possibly originates from the regulation of G1 to S phase transcriptional network.

The results of wound healing and transwell assays show that silencing ELMO3 significantly suppressed the migration and invasion of HCT116 cells. These results indicate ELMO3 participates in the process of CRC invasion and metastasis. This is in agreement with a previous report, where ELMO3 overexpression could promote the migration of lung tumor cells through the blood stream. A previous research showed that the ELMO3 gene can serve as a target gene of caudal-related homeobox transcription factor 2 (CDX2) in colorectal Caco-2 cells [36]. As an intestine-specific transcription factor, CDX2 can modulate cellular processes such as cell differentiation, proliferation, cell adhesion, migration, and tumorigenesis [40]. Therefore, the process of ELMO3 modulating cell invasion and migration is likely controlled by CDX2.

A previous study showed that ELMO1 plays important roles in cytoskeleton rearrangements during cellular migration in *C. elegans* [11]. The cytoskeleton possesses the ability to rapidly and substantially remodel the cell. F-actin polymerization is necessary for cytoskeleton rearrangement. Our result shows that that knockdown of ELMO3 inhibited F-actin polymerization, and it proves that ELMO3 can manipulate the migration of HCT116 cells and has a similar function in cell motility as ELMO1. In breast cancer, binding of the chemokine CXCL12 and its G-protein-coupled receptor CXCR4 triggers Gαi2 and promotes its association with the ELMO1/Dock180 complex, which promotes the membrane translocation and activation of Rac1 and Rac2, and thereby contributes to actin cytoskeleton changes during breast cancer metastasis [18]. In addition, another report found that Dock1 (Dock 180) promotes heregulin-mediated Rac activation, which is essential for breast cancer growth and metastasis [41]. However, breast cancer cells do not express ELMO3. A further investigation is necessary to explore how ELMO3 leads to colorectal cancer cell migration through regulating actin polymerization and the cytoskeleton.

In conclusion, the results of our study show that the ELMO3 protein as a potential oncogene was overexpressed in CRC tumor tissues. Its expression was correlated with clinicopathological characteristics, including tumor size, tumor differentiation, TNM stage, lymph node metastasis and distant metastasis. Furthermore, ELMO3 can act as a critical regulator in proliferation, invasion and metastasis. It is expected to become a novel biomarker of diagnosis and a therapy target of CRC. The related regulatory mechanism deserves further exploration.

## 4. Materials and Methods

### 4.1. Patients and Samples

This study was conducted in accordance with the Declaration of Helsinki. The protocol was approved by the Ethics Committee of the Second Affiliated Hospital of Nanchang University (No. 2014020, 20/10/2014), and written informed consent was obtained from all participants. A total of 45 cases of paired tumor tissues and adjacent normal tissues were obtained from patients undergoing surgery in the Second Affiliated Hospital of Nanchang University from January 2015 to March 2016. The adjacent normal tissues were taken 5 cm laterally from the edge of the cancerous region. None of the patients had a history of previous therapies with antitumor drugs or radiotherapy prior to operation.

All case samples were excised in three parts: two parts were immediately frozen in liquid nitrogen following surgical resection for mRNA and protein extraction, and the third one was fixed in 10% formalin, embedded in paraffin and then cut into 4 µm sections for IHC analysis. The samples were obtained from 18 female and 27 male subjects, with a mean age of 59.2 years (range 20–88). Among them, 11 cases presented with lymph node metastasis, 33 cases lacked lymph node metastasis and one case could not be confirmed. Furthermore, 8, 28 and 9 cases displayed high, moderate and low differentiation, respectively. The clinicopathologic characteristics, including age, gender, tumor size, differentiation status, TNM stage, lymph node metastasis, and distant metastasis were recorded in a database (Table 2).

### 4.2. RT-PCR and qPCR Analysis

Total RNA was extracted from CRC tissues and cells using TRIzol reagent (Invitrogen Life Technologies, Carlsbad, CA, USA). The total RNA concentration was determined, and then 1 μg of total RNA was reverse transcribed using the Two-Step kit (Promega Corporation, Madison, WI, USA) according to the manufacturer’s instructions. The qPCR was performed on a CFX96 Real-Time system machine (Bio-Rad, Hercules, CA, USA) with GAPDH as a control. The following primers were used to amplify target sequences: ELMO3, forward 5’-ACCAATGGGCGACGAGAT-3’ and reverse 5’-TGCTGGGTTGCTGTTAGA-3’, 250 bp (GenScript USA Inc., Nanjing, China); GAPDH, forward 5’-TGACTTCAACAGCGACACCCA-3’ and reverse 5’-CACCCTGTTGCTGTAGCCAAA-3’, 121 bp (GenScript USA Inc, Nanjing, China). All amplifications were performed in the final reaction mixture (20 μL) and in triplicate. The relative change of mRNA transcriptional level was calculated using the 2^−ΔΔ*C*t^ method [42].

### 4.3. Western Blot Analysis

To detect the protein level of ELMO3 in CRC tissues and cells, Western blotting was carried out as previously reported [43,44]. Briefly, 25 μg of total protein was separated on a 10% SDS-PAGE gel and then transferred electrophoretically onto a 0.45 μm PVDF membrane (Millipore, Billerica, MA, USA), followed by immunoblotting with a polyclonal primary antibody against ELMO3 (1:125; Thermoscientific, Rockford, IL, USA) overnight at 4 °C. A human anti-β-actin monoclonal antibody (1:1000; ComWin Biotech Co., Ltd., Beijing, China) was used as an internal control. The membrane was further incubated for 2 h with goat anti-mouse IgG conjugated with peroxidase (Beyotime, Beijing, China). An Imagelab Analysis System machine (Bio-Rad ChemiDoc MP Imaging System, Hercules, CA, USA) was used to detect the expression level of the target protein. The band intensities were quantified using Image-Pro Plus 6.0 software (Media Cybernetics, Silver Spring, MD, USA).

### 4.4. Immunohistochemical Analysis

The IHC analysis was performed on paraffin-embedded sections using a Streptavidin-Biotin Complex (SABC) procedure. Briefly, antigen retrieval was performed by heating the deparaffinized, rehydrated sections in 0.01 M citrate buffer (pH 6.0) for 20 min, followed by blocking with 10% normal rabbit serum. Subsequently, the tissue slides were incubated overnight at 4 °C with the primary antibody (goat anti human-ELMO3 polyclonal antibody, Thermo Scientific, Rockford, IL, USA) at a final dilution of 1:100. For the negative control samples, phosphate buffered saline (PBS) instead of primary antibodies was used for incubation. After incubation, the sections were washed three times in PBS, and then incubated with a biotinylated secondary antibody for 30 min and then incubated with a ready-to-use SABC-POD (goat IgG) kit (Boster Biological Engineering Co., Ltd., Wuhan, China) for 30 min. The sample slides were then counterstained using hematoxylin. Finally, the immunostained slides were reviewed in a blind manner by two pathologists who did not know the clinical pathological features of the patients. The expression levels of ELMO3 were judged based on both the intensity of staining and the proportion of positively stained tumor cells following the examination of ≥500 cells in five representative areas. The cells at each intensity of staining were recorded as follows: 0, no staining; 1, weak staining; 2, moderate staining; and 3, strong staining. The proportion of stained tumor cells that were positive for ELMO3 expression was graded as follows: <5% (score 0), 5%–25% (score 1), 25%–50% (score 2), 50%–75% (score 3), and >75% (score 4). The final scores were calculated by multiplying the intensity score to the percentage score. A final score of 0–1 was defined as (−), while scores of 2–4, 5–8 and 9–12 were considered (+), (++) and (+++), respectively. The positive expression rate (%) = the cases of positive expression (weak + moderate + strong) accounted for all cases × 100%.

### 4.5. Cell Culture and ELMO3 siRNA Transfection

The human CRC cell lines HCT116, LoVo, COLO205, HT29, SW480 and SW620 were purchased from the Type Culture Collection of the Chinese Academy of Sciences (Shanghai, China). The cells were cultured in RPMI 1640 medium (Solarbio, Beijing, China) with 10% fetal bovine serum (FBS) (Hyclone, Logan, UT, USA) and 100 U/mL penicillin and incubated at 37 °C with 5% CO_2_. To detect the effects of ELMO3 knockdown on invasion and metastasis in the poorly differentiated HCT116 cells, three different small interfering RNAs (siRNAs) targeting the ELMO3 RNA and an NC siRNA were designed and synthesized by GenePharma (Shanghai, China). Then, HCT116 cells were transfected with these siRNAs and controls using LipofectamineTM 2000 reagent (Invitrogen, Carlsbad, CA, USA) according to the manufacturer’s instructions. After 24 and 48 h of transfection, the transfection and knockdown efficiency of the ELMO3 siRNA were detected by Western blot analysis.

### 4.6. MTS Assay

The effect of silencing ELMO3 on cell viability was detected by MTS assay according to the manufacturer’s protocol (CellTiter 96 Aqueous One Solution Cell Proliferation assay; Promega, Madison, WI, USA). After 24 h of transfection, HCT116 cells were plated in 96-well plates at a density of 5 × 10^2^/100 µL per well. After 24, 48, 72, 96 and 120 h of incubation, 20 µL of the MTS assay solution was added to each well and incubated at 37 °C for 3 h. Cell viability was calculated by measuring the absorbance at 490 nm. The experiments were repeated three times.

### 4.7. Cell Cycle Analysis

The effect of ELMO3 knockdown on the cell cycle regualtion of HCT116 cells was examined by flow cytometry. HCT116 cells were seeded in a 12-well plate at a density of 1.5 × 10^5^ cells per well overnight. After 24 h of transfection, the harvested cells were fixed in 70% ice-cold ethanol and stored at 4 °C overnight. The fixed cells were washed twice with PBS and then incubated with 500 µL of PI (20 µg/mL) (Sigma, St. Louis, MO, USA) staining solution in the dark at room temperature for 30 min. The staining solution contained 0.1% Triton X-100 (Sigma, St. Louis, MO, USA) and RNase A (Sigma). The DNA contents of cells were qualified using a BD FACSCalibur flow cytometer (Becton Dickinson, San Jose, CA, USA).

### 4.8. Wound Healing Assays 

A wound healing assay was used to evaluate the tumor cell motility capacity in vitro. After 24 h of transfection, 5 × 10^5^ cells per well were grown to 80%–90% confluency in a 12-well plate. The monolayer was then scratched using a sterile 10 μL pipette tip. After the removal of the detached cells by gentle washing with PBS, the cells were fed with fresh complete medium and incubated at 37 °C to allow the cells to migrate into the open wounds. Three photographs of randomly selected wound areas were taken within appropriate time points (0, 24 and 48 h) under a light microscope (Olympus Corp., Tokyo, Japan). The experiments were performed in triplicate, and representative images were analyzed by ImageJ software.

### 4.9. Transwell Migration and Invasion Assay

To perform the migration assay, 5 × 10^4^ cells per well were seeded into the upper chambers of Transwell filter (Corning Costar, Acton, MA, USA) with serum-free RPMI 1640 medium. To conduct the invasion assay, 1 × 10^5^ cells were seeded in the upper chambers of the filters coated with 1 mg/mL Matrigel (BD Biosciences, San Jose, CA, USA). Then, 600 µL of RPMI-1640 medium containing 20% FBS was added to the lower chamber. After 12, 24 and 48 h of incubation, to examine migration and invasion, the non-migrated cells on the upper surface of the membrane were removed, and the invading cells below the membrane were fixed with 4% paraformaldehyde for 20 min and stained with hematoxylin-eosin (HE) at 37 °C for 15 min. Under a 100× inverted microscope, five fields on each membrane were randomly selected, and the mean number of penetrating cells was calculated.

### 4.10. Immunofluorescence Staining

Cells (5 × 10^4^) were seeded in 24-well plates containing sterile coverslips. After transfection with siRNA and culture for 24 h, the cells were fixed with 10% paraformaldehyde for 10 min, permeabilized in 0.1% TritonX-100 in PBS for 5 min and blocked in 1% BSA for 20 min. The cells were then stained with 1 mM FITC-phalloidin (Sigma) for 40 min in the dark at room temperature to visualize the actin cytoskeleton, and counterstained with 50 mM DAPI (Boster, Wuhan, China) for 2 min in the dark at room temperature to visualize cell nuclei. The cells were washed in PBS to remove the unbound staining dyes. Finally, the morphological characteristics of the cytoskeleton and nucleus were observed using a fluorescence microscope (Nikon Eclipseni NI, Tokyo, Japan). The colocalization efficiency was calculated using ImageJ software (NIH, Bethesda, MD, USA). Twenty-five images were analyzed.

### 4.11. Statistical Analysis

Statistical analysis was performed using SPSS standard version 11.5 (SPSS Inc., Chicago, IL, USA) software. All data are presented as the mean ± standard deviation (SD). The statistical significance for comparisons between groups was determined using Student’s t-test or an ANOVA. The nonparametric Mann–Whitney U test and Kruskal–Wallis H test were applied to analyze the relationship between ELMO3 protein expression and the clinicopathologic characteristics of CRC. All *p* values were two-tailed, and differences with *p* < 0.05 were considered statistically significant.

## Figures and Tables

**Table 1:** Statistics of staining scores of ELMO3 in CRC tissues.

Groups	N	ELMO3 Immunoreactivity Score				Positive Rate (%)
		−	+	++	+++	
Tumor	45	5	9	12	19	88.89% **
Normal	45	28	9	6	2	37.78%

** *p* < 0.01.

**Table 2:** Association analysis of ELMO3 expression with clinicopathological parameters of CRC patients.

Clinical Parameter	ELMO3 Immunoreactivity Score				N	*p*-Value
	−	+	++	+++		
Gender						0.883 ^†^
Male	5	5	4	13	27	
Female	0	4	8	6	18	
Age (years)						
≤60	3	6	9	7	25	0.094 ^†^
>60	2	3	3	12	20	
Tumor size (cm)						0.007 ^†^
≤4.0	4	8	7	7	26	
>4.0	1	1	5	12	19	
Differentiation						0.001 ^‡^
High	4	3	1	0	8	
Moderate	1	5	10	12	28	
Poor	0	1	1	7	9	
Depth of invasion						0.009 ^†^
T1 + T2	4	1	4	1	10	
T3 + T4	1	8	8	18	35	
Lymph node metastasis						0.003 ^†^
No	5	9	9	10	33	
Yes	0	0	3	9	12	
Distant metastasis						0.013 ^†^
No	5	9	12	14	40	
Yes	0	0	0	5	5	
TNM classification						0.000 ^†^
I + II	5	9	9	7		
III + IV	0	0	3	11		
Lymph node positive number						0.003 ^†^
<1	5	9	9	10	33	
≥1	0	0	3	9	12	

^†^ statistical analysis was performed by the Mann–Whitney U test; ^‡^ statistical analysis was performed by Kruskal–Wallis H test.

## Supplementary Materials

Supplementary materials can be found at www.mdpi.com/1422-0067/17/12/2119/s1.

## References

[1] Anitha A., Maya S., Sivaram A.J., Mony U., Jayakumar R. (2016). Combinatorial nanomedicines for colon cancer therapy. Wires Nanomed. Nanobiotechnol..

[2] Cunningham D., Atkin W., Lenz H.-J., Lynch H.T., Minsky B., Nordlinger B., Starling N. (2010). Colorectal cancer. Lancet.

[3] Lin C.C., Ng H.L.H., Pan W.S., Chen H.B., Zhang G., Bian Z.X., Lu A.P., Yang Z.J. (2015). Exploring different strategies for efficient delivery of colorectal cancer therapy. Int. J. Mol. Sci..

[4] Brody H. (2015). Colorectal cancer. Nature.

[5] Chen W., Zheng R., Baade P.D., Zhang S., Zeng H., Bray F., Jemal A., Yu X.Q., He J. (2016). Cancer statistics in China. CA Cancer J. Clin..

[6] Kanas G.P., Taylor A., Primrose J.N., Langeberg W.J., Kelsh M.A., Mowat F.S., Alexander D.D., Choti M.A., Poston G. (2012). Survival after liver resection in metastatic colorectal cancer: Review and meta-analysis of prognostic factors. Clin. Epidemiol..

[7] Nicolson G.L. (1993). Paracrine and autocrine growth mechanisms in tumor metastasis to specific sites with particular emphasis on brain and lung metastasis. Cancer Metastasis Rev..

[8] Zlotnik A. (2006). Involvement of chemokine receptors in organ-specific metastasis. Contrib. Microbiol..

[9] Iijima M., Devreotes P. (2002). Tumor suppressor PTEN mediates sensing of chemoattractant gradients. Cell.

[10] Yan J., Mihaylov V., Xu X., Brzostowski J.A., Li H., Liu L., Veenstra T.D., Parent C.A., Jin T. (2012). A Gβγ effector, ELMOE, transduces GPCR signaling to the actin network during chemotaxis. Dev. Cell.

[11] Gumienny T.L., Brugnera E., Tosello-Trampont A.C., Kinchen J.M., Haney L.B., Nishiwaki K., Walk S.F., Nemergut M.E., Macara I.G., Francis R. (2001). CED-12/ELMO, a novel member of the CrkII/Dock180/Rac pathway, is required for phagocytosis and cell migration. Cell.

[12] Geisbrecht E.R., Haralalka S., Swanson S.K., Florens L., Washburn M.P., Abmayr S.M. (2008). Drosophila ELMO/CED-12 interacts with Myoblast city to direct myoblast fusion and ommatidial organization. Dev. Biol..

[13] Brzostowski J.A., Fey P., Yan J., Isik N., Jin T. (2009). The ELMO family forms an ancient group of actin-regulating proteins. Commun. Integr. Biol..

[14] Patel M., Pelletier A., Cote J.F. (2011). Opening up on ELMO regulation: New insights into the control of Rac signaling by the DOCK180/ELMO complex. Small GTPases.

[15] Patel M., Margaron Y., Fradet N., Yang Q., Wilkes B., Bouvier M., Hofmann K., Cote J.F. (2010). An evolutionarily conserved autoinhibitory molecular switch in ELMO proteins regulates Rac signaling. Curr. Biol..

[16] Jarzynka N.J., Hu B., Hui K.M., Bar-Joseph I., Gu W.S., Hirose T., Haney L.B., Ravichandran K.S., Nishikawa R., Cheng S.Y. (2007). ELMO1 and Dock180, a bipartite Rac1 guanine nucleotide exchange factor, promote human glioma cell invasion. Cancer Res..

[17] Zhang B., Shi L., Lu S., Sun X., Liu Y., Li H., Wang X., Zhao C., Zhang H., Wang Y. (2015). Autocrine IL-8 promotes F-actin polymerization and mediate mesenchymal transition via ELMO1-NF-κB-Snail signaling in glioma. Cancer Biol. Ther..

[18] Li H., Yang L., Fu H., Yan J., Wang Y., Guo H., Hao X., Xu X., Jin T., Zhang N. (2013). Association between Galphai2 and ELMO1/Dock180 connects chemokine signalling with Rac activation and metastasis. Nat. Commun..

[19] Jiang J.R., Liu G.Q., Miao X.Y., Hua S.W., Zhong D.W. (2011). Overexpression of engulfment and cell motility 1 promotes cell invasion and migration of hepatocellular carcinoma. Exp. Ther. Med..

[20] Wang J., Dai J.M., Che Y.L., Gao Y.M., Peng H.J., Liu B., Wang H., Hua L.H. (2014). ELMO1 Helps Dock180 to Regulate Rac1 Activity and Cell Migration of Ovarian Cancer. Int. J. Gynecol. Cancer.

[21] Wang H., Linghu H., Wang J., Che Y.L., Xiang T.X., Tang W.X., Yao Z.W. (2010). The role of Crk/Dock180/Rac1 pathway in the malignant behavior of human ovarian cancer cell SKOV3. Tumor Biol..

[22] Dulak A.M., Stojanov P., Peng S.Y., Lawrence M.S., Fox C., Stewart C., Bandla S., Imamura Y., Schumacher S.E., Shefler E. (2013). Exome and whole-genome sequencing of esophageal adenocarcinoma identifies recurrent driver events and mutational complexity. Nat. Genet..

[23] Rapa E., Hill S.K., Morten K.J., Potter M., Mitchell C. (2012). The over-expression of cell migratory genes in alveolar rhabdomyosarcoma could contribute to metastatic spread. Clin. Exp. Metastasis.

[24] Stevenson C., de la Rosa G., Anderson C.S., Murphy P.S., Capece T., Kim M., Elliott M.R. (2014). Essential role of ELMO1 in Dock2-dependent lymphocyte migration. J. Immunol..

[25] Wang Y., Xu X., Pan M., Jin T. (2016). ELMO1 Directly Interacts with Gβγ Subunit to Transduce GPCR Signaling to Rac1 Activation in Chemotaxis. J. Cancer.

[26] Epting D., Slanchev K., Boehlke C., Hoff S., Loges N.T., Yasunaga T., Indorf L., Nestel S., Lienkamp S.S., Omran H. (2015). The Rac1 regulator ELMO controls basal body migration and docking in multiciliated cells through interaction with Ezrin. Development.

[27] Franke K., Otto W., Johannes S., Baumgart J., Nitsch R., Schumacher S. (2012). miR-124-regulated RhoG reduces neuronal process complexity via ELMO/Dock180/Rac1 and Cdc42 signalling. EMBO J..

[28] East M.P., Bowzard J.B., Dacks J.B., Kahn R.A. (2012). ELMO Domains, Evolutionary and Functional Characterization of a Novel GTPase-activating Protein (GAP) Domain for ARF Protein Family GTPases. J. Biol. Chem..

[29] Grimsley C.M., Kinchen J.M., Tosello-Trampont A.C., Brugnera E., Haney L.B., Lu M., Chen Q., Klingele D., Hengartner M.O., Ravichandran K.S. (2004). Dock180 and ELMO1 proteins cooperate to promote evolutionarily conserved Rac-dependent cell migration. J. Biol. Chem..

[30] Toret C.P., Collins C., Nelson W.J. (2014). An ELMO-Dock complex locally controls Rho GTPases and actin remodeling during cadherin-mediated adhesion. J. Cell Biol..

[31] Yang L. (2013). The Functions of ELMO2 on Hepatocellular Carcinoma Cells Migration and Invasion. Master’s Thesis.

[32] Fan W., Yang H.K., Xue H., Sun Y., Zhang J. (2015). ELMO3 is a novel biomarker for diagnosis and prognosis of non-small cell lung cancer. Int. J. Clin. Exp. Pathol..

[33] Soes S., Daugaard I.L., Sorensen B.S., Carus A., Mattheisen M., Alsner J., Overgaard J., Hager H., Hansen L.L., Kristensen L.S. (2014). Hypomethylation and increased expression of the putative oncogene ELMO3 are associated with lung cancer development and metastases formation. Oncoscience.

[34] Kristensen L.S., Soes S., Hansen L.L. (2014). ELMO3: A direct driver of cancer metastasis?. Cell Cycle.

[35] Goyette M.A., Cote J.F. (2014). NSCLC metastasis: Going with ELMO3. Oncotarget.

[36] Coskun M., Boyd M., Olsen J., Troelsen J.T. (2010). Control of Intestinal Promoter Activity of the Cellular Migratory Regulator Gene ELMO3 by CDX2 and SP1. J. Cell. Biochem..

[37] Olson M.F., Sahai E. (2009). The actin cytoskeleton in cancer cell motility. Clin. Exp. Metastasis.

[38] Abu-Thuraia A., Gauthier R., Chidiac R., Fukui Y., Screaton R.A., Gratton J.P., Cote J.F. (2015). Axl phosphorylates ELMO scaffold proteins to promote Rac activation and cell invasion. Mol. Cell. Biol..

[39] Duronio R.J., Xiong Y. (2013). Signaling pathways that control cell proliferation. Cold Spring Harb. Perspect. Biol..

[40] Coskun M., Troelsen J.T., Nielsen O.H. (2011). The role of CDX2 in intestinal homeostasis and inflammation. Biochim. Biophys. Acta.

[41] Laurin M., Huber J., Pelletier A., Houalla T., Park M., Fukui Y., Haibe-Kains B., Muller W.J., Cote J.F. (2013). Rac-specific guanine nucleotide exchange factor DOCK1 is a critical regulator of HER2-mediated breast cancer metastasis. Proc. Natl. Acad. Sci. USA.

[42] Livak K.J., Schmittgen T.D. (2001). Analysis of relative gene expression data using real-time quantitative PCR and the 2**^−^**^ΔΔ*C*t^ Method. Methods.

[43] Yu Q., Shen W., Zhou H., Dong W., Gao D. (2016). Knockdown of LI-cadherin alters expression of matrix metalloproteinase-2 and -9 and galectin-3. Mol. Med. Rep..

[44] Gao D., Xu Z., Kuang X., Qiao P., Liu S., Zhang L., He P., Jadwiga W.S., Wang Y., Min W. (2014). Molecular characterization and expression analysis of the autophagic gene Beclin 1 from the purse red common carp (*Cyprinus carpio*) exposed to cadmium. Comp. Biochem. Physiol. C Toxicol. Pharmacol..

