# Supplementary material for: Knockdown of ELMO3 Suppresses Growth, Invasion and Metastasis of Colorectal Cancer
Source: Int J Mol Sci. 2016 Dec 16;17(12):2119. doi: 10.3390/ijms17122119 (PMC5187919; doi:10.3390/ijms17122119)
Supplement: Supplementary file 1 [file ijms-17-02119-s001.pdf]

Supplementary Materials: Knockdown of ELMO3 Suppresses Growth, Invasion and Metastasis of Colorectal Cancer

Hui-Yun Peng, Qiong-Fang Yu, Wei Shen, Cheng-Ming Guo, Zhen Li, Xiao-Yan Zhou, Nan-Jin Zhou, Wei-Ping Min and Dian Gao

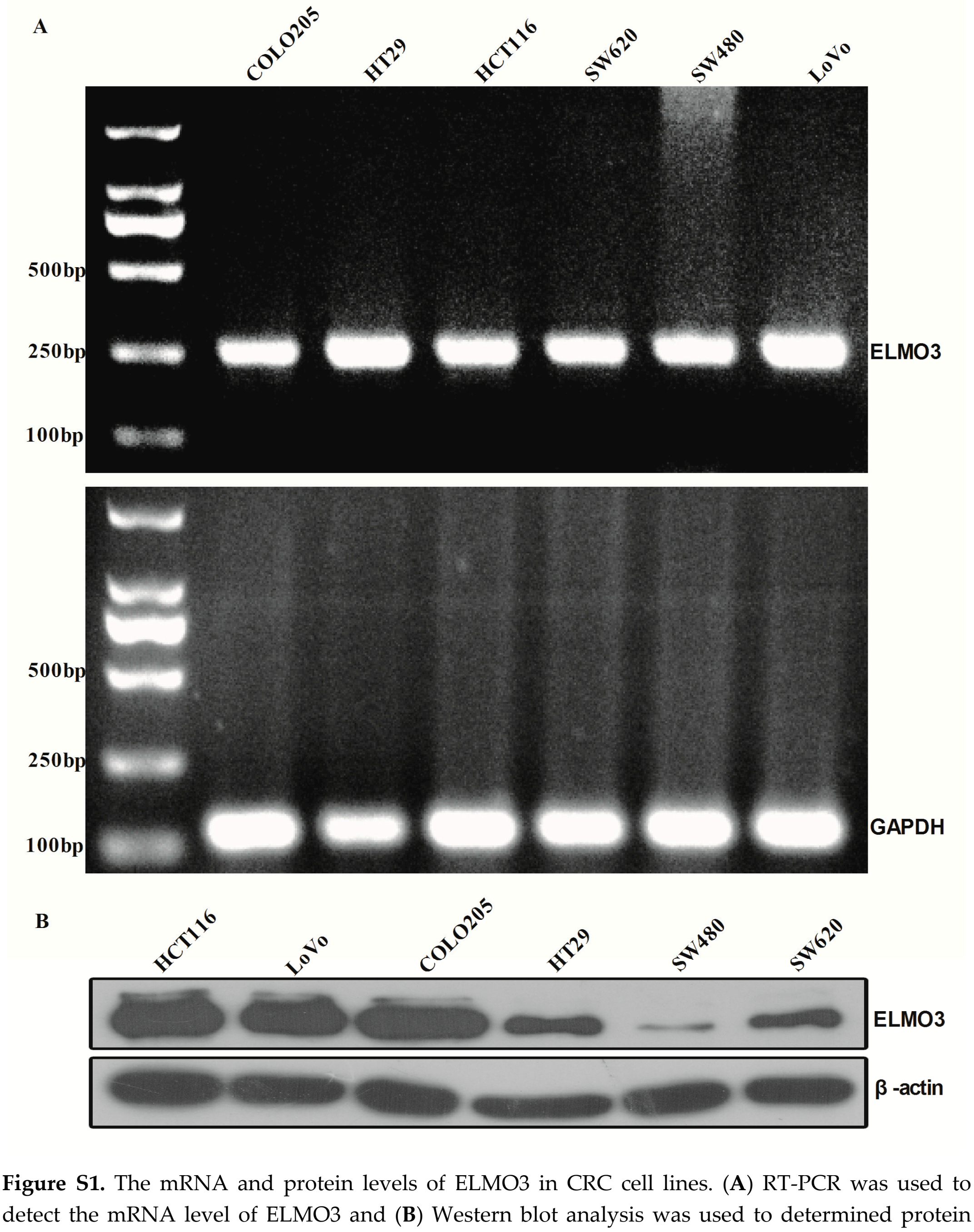

Figure S1. The mRNA and protein levels of ELMO3 in CRC cell lines. (**A**) RT-PCR was used to detect the mRNA level of ELMO3 and (**B**) Western blot analysis was used to determined protein level of ELMO3.
